# Case Report: Amiodarone-triggered refractory ventricular fibrillation storm in septic cardiomyopathy with pre-existing QT prolongation: termination using lidocaine and anisodamine

**DOI:** 10.3389/fmed.2026.1841318

**Published:** 2026-06-10

**Authors:** Min Wu, Lutao Xie, Pin Lan, Meisong Chen

**Affiliations:** Department of Emergency Medicine, Lishui Central Hospital, The Fifth Affiliated Hospital of Wenzhou Medical University, Lishui, China

**Keywords:** anisodamine, case report, lidocaine, QTc interval, septic cardiomyopathy (SCM), torsades de pointes (TdP), ventricular fibrillation (VF)

## Abstract

**Background:**

Septic cardiomyopathy (SCM) is a reversible acute cardiac dysfunction caused by sepsis. The use of QT-prolonging agents such as moxifloxacin and amiodarone may induce life-threatening arrhythmias, including torsades de pointes (TdP) and ventricular fibrillation (VF), both associated with high morbidity and mortality, posing significant challenges in clinical management.

**Case presentation:**

We report the case of an 89-year-old male who developed septic cardiomyopathy secondary to enterogenic septic shock, presenting with marked corrected QT (QTc) prolongation and impaired cardiac function. Following intravenous administration of moxifloxacin and amiodarone, QTc interval further prolonged, leading to frequent premature ventricular contractions that progressed to refractory TdP and VF. Despite conventional treatment, including electrolyte correction, defibrillation, and cardiopulmonary resuscitation, the arrhythmias remained uncontrollable. Intravenous maintenance therapy with lidocaine combined with anisodamine was initiated, resulting in gradual resolution of arrhythmias. No further TdP or VF episodes occurred. After 2 weeks, left ventricular function returned to baseline, and the patient was discharged in stable condition. A 9-month follow-up revealed near-complete recovery of cardiac function.

**Conclusion:**

Moxifloxacin and amiodarone may trigger refractory TdP and VF in patients with septic cardiomyopathy. Intravenous lidocaine combined with anisodamine may be a potential therapeutic option for terminating these refractory arrhythmias.

## Background

Septic cardiomyopathy (SCM) is a reversible form of acute myocardial dysfunction induced by sepsis, typically presenting as transient biventricular dilation, decreased ejection fraction, and reduced myocardial contractility. Despite its reversible nature, the condition carries a mortality rate as high as 70%–90%, posing a significant clinical challenge to critical care management (1, 2). TdP is a polymorphic ventricular tachycardia closely associated with QT/QTc interval prolongation. Without prompt recognition and treatment, TdP may degenerate into VF, leading to sudden cardiac death. In septic patients, the risk of QTc prolongation is heightened by systemic inflammatory responses and the use of QT-prolonging medications (3). Moxifloxacin and amiodarone are widely used in clinical settings but are known to prolong the QT interval and can precipitate TdP. We present a case of an 89-year-old man with septic shock who developed refractory TdP and VF after receiving moxifloxacin and amiodarone. The arrhythmias were unresponsive to standard resuscitative measures, including defibrillation, magnesium/potassium repletion, and cardiopulmonary resuscitation. Ultimately, intravenous lidocaine combined with anisodamine successfully terminated the arrhythmias. This case highlights the potential value of this therapeutic combination in high-risk patients with SCM and malignant arrhythmias.

## Case presentation

An 89-year-old male presented to the emergency department with a 3-day history of diarrhea and fever and a 2-day history of confusion. He had a past medical history of benign prostatic hyperplasia. He reported ingesting leftover food 3 days prior to admission, after which he developed yellow, watery stools more than 10 times per day, accompanied by chills and fever. Two days before admission, he developed profuse sweating, cold extremities, and altered consciousness. Upon arrival at the resuscitation room, the patient presented with high fever and worsening disturbance of consciousness. His body temperature was 39.7 °C, blood pressure was 79/43 mmHg, respiratory rate was 28 breaths per minute, and heart rate was 67 beats per minute with an irregular rhythm. The abdomen was soft, without tenderness or rebound pain, and bowel sounds were hyperactive. Laboratory results showed: Procalcitonin (PCT), 64.11 ng/mL; C-reactive protein (CRP), 99.06 mg/L; white blood cell count (WBC), 13.0 × 10^9^/L; Platelets (PLT), 22 × 10^9^/L; troponin I (TnI), 6.14 ng/mL; Myoglobin (MYO), 1016.0 ng/mL; B-type natriuretic peptide (BNP), 2801.5 pg/mL; Potassium (K^+^), 3.8 mmol/L; electrocardiogram (ECG): sinus rhythm, ST depression, T wave inversion, QTc interval, 514 ms; echocardiogram, Left Ventricular Ejection Fraction (LVEF) 69%, no segmental wall motion abnormalities; Abdominal CT, mild ascites; quick Sequential Organ Failure Assessment score was 3. The initial diagnoses included septic shock, acute gastroenteritis, myocardial injury, acute heart failure, and thrombocytopenia. After admission, the patient was started on intravenous moxifloxacin (0.4 g daily) and imipenem/cilastatin (1 g every 8 h), norepinephrine (0.1–0.6 μg/kg/min), recombinant human thrombopoietin (15,000 U SC daily), fresh plasma, and K^+^ supplementation. Other supportive medications included rabeprazole sodium enteric-coated tablets, live *Clostridium butyricum* capsules, and vitamin C.

**FIGURE 1:**
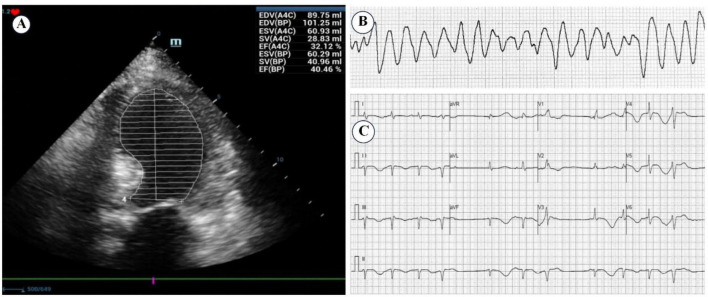
**(A)** Transthoracic echocardiogram on day 4 showing near-complete loss of wall motion in the left ventricular papillary muscle segments, impaired diastolic function, and a Left Ventricular Ejection Fraction (LVEF) of 40%. **(B)** Electrocardiogram on day 5 demonstrating torsades de pointes (TdP) and ventricular fibrillation (VF). **(C)** Electrocardiogram on day 5 after administration of lidocaine combined with anisodamine showing restoration of sinus rhythm, with a corrected QT (QTc) interval of 647 ms.

On day 4, blood cultures returned positive for *Escherichia coli*. The patient developed frequent premature ventricular contractions (PVCs), First-degree atrioventricular block, and QTc prolongation (500 ms, calculated using the Bazett formula). Echocardiogram revealed markedly reduced wall motion of the papillary muscle segments and an LVEF of 40% (Figure 1A). Seven hours after moxifloxacin infusion (at 19:48 on Day 4 of admission), the patient developed TdP followed by VF. Immediate cardiopulmonary resuscitation (CPR) was initiated, along with biphasic unsynchronized defibrillation (200 J every 2 min) and epinephrine (1 mg every 3 min). After 12 min, sinus rhythm was restored, but frequent PVCs persisted. Therefore, after return of spontaneous circulation, Amiodarone (150 mg IV bolus over 10 min followed by 1 mg/min continuous infusion) was administered, and the patient was intubated and transferred to the emergency intensive care unit (EICU).

**FIGURE 2:**
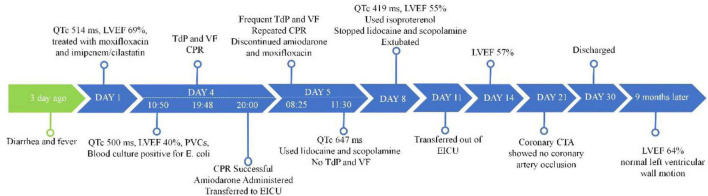
Patient’s treatment course.

Upon arrival at the EICU, the patient had frequent episodes of ventricular tachycardia. Follow-up laboratory results showed: PCT, 23.74 ng/mL; CRP, 75.78 mg/L; WBC, 4.3 × 10^9^/L; PLT, 16 × 10^9^/L; TnI, 9.05 ng/mL; MYO, 175.8 ng/mL; BNP, 2279.7 pg/mL; K^+^, 3.41 mmol/L; Magnesium (Mg^2+^): 0.75 mmol/L (reference range: 0.75–1.02 mmol/L). The cardiology department was consulted for assistance. Given the patient’s markedly prolonged QTc interval, moxifloxacin and amiodarone were discontinued. The patient received intravenous lidocaine (100 mg), electrolyte replacement, sedation, and analgesia. However, he developed recurrent, refractory TdP and VF that could not be controlled despite multiple cycles of CPR and defibrillation (Figure 1B). A continuous infusion of lidocaine (120 mg/h) and anisodamine (2 mg/h) was started. Arrhythmias were gradually suppressed, with no further episodes of TdP or VF. Post-resuscitation laboratory results showed: K^+^, 4.01 mmol/L; Mg^2+^, 0.94 mmol/L; ECG: sinus rhythm at 80 bpm, second-degree atrioventricular block (Mobitz type I), QTc interval, 647 ms (Figure 1C). By day 8, the patient regained consciousness, with blood pressure 154/65 mmHg. Echocardiogram showed improved wall motion and LVEF 55%. ECG showed QTc of 419 ms with bradycardia (30–50 bpm) and arrhythmia. Isoproterenol was administered for rate control, and lidocaine and anisodamine were discontinued. The patient was extubated and transferred to cardiology. Final diagnoses included post-cardiac arrest, septic shock, septic cardiomyopathy, malignant arrhythmia, and QT prolongation. On day 14, echocardiogram revealed normal wall motion and LVEF 57%. Coronary CTA performed on day 22 showed no obstructive coronary disease. At 9-month follow-up, echocardiogram demonstrated normal left ventricular function (LVEF 64%). The patient successfully underwent transurethral prostate surgery under general anesthesia and returned to normal social life. Table 1 provides detailed blood test results and QTc interval changes before extubation. The treatment process is summarized in Figure 2.

**TABLE 1:** Patient’s blood tests and corrected QT (QTc) interval changes.

Time	CRP (mg/L)	WBC (×10^9^/L)	PCT (ng/mL)	TnI (ng/L)	MYO (ng/mL)	BNP (pg/mL)	K^+^ (mmol/L)	QTc (ms)
Day 1	99.06	13	64.11	6.14	1016	2801.5	3.8	514
Day 2	75.78	4.3	23.74	9.05	175.8	2279.7	3.41	–
Day 4	27.38	6.7	1.46	2.12	133.4	1715.4	3.1	500
Day 5	61.91	8.2	1.03	1.97	77.5	3271.7	4.03	647
Day 8	212.86	10.6	0.67	1.72	84.1	778.8	4.02	419

“–” indicates that the corrected QT (QTc) interval was not measured on that day.

## Discussion

Sepsis is a systemic inflammatory response syndrome triggered by infection, which, in severe cases, may lead to organ dysfunction or multiorgan failure. As sepsis progresses, myocardial dysfunction becomes a significant contributor to patient deterioration (4). SCM is a reversible form of acute myocardial depression unrelated to myocardial ischemia. It typically manifests as transient ventricular dilation, reduced ejection fraction, and impaired systolic function. The incidence of SCM varies between 10% and 70%, with reported mortality rates reaching 70%–90% (1, 2). To date, there is no universally accepted diagnostic standard for SCM. Early diagnostic criteria were based on new-onset EF reduction to ≤50% or a ≥10% decrease from baseline in the presence of confirmed sepsis. More recent evidence supports the use of speckle-tracking echocardiography to identify impaired myocardial contractility for diagnosis (5). The pathophysiology of SCM is complex and multifactorial, involving immune-inflammatory imbalance, calcium-handling abnormalities, mitochondrial dysfunction, apoptosis, and coronary microcirculatory endothelial injury (6). Unlike ischemic cardiomyopathy, SCM does not result from coronary artery obstruction, though its precise mechanisms remain incompletely understood (7).

Torsades de pointes is a rare but potentially fatal polymorphic ventricular tachycardia closely associated with QT/QTc prolongation. It is typically drug-induced and may degenerate into VF, resulting in sudden cardiac death if not promptly managed (8, 9). A QTc interval exceeding 500 ms is considered markedly abnormal and associated with a high risk of malignant arrhythmia (10). Intervention is recommended when QTc is ≥470–500 ms in men, ≥480–500 ms in women, or increases by ≥60 ms from baseline (11). Moxifloxacin, a fourth-generation 8-methoxy fluoroquinolone, is commonly used in clinical practice but has been linked to cardiovascular adverse events including QT prolongation and valvular toxicity (12). In healthy subjects, moxifloxacin prolongs QTc by approximately 11.5–19.5 ms, while in elderly pneumonia patients, QTc prolongation ≥30–60 ms occurs in up to 15.5% of cases (13). The patient’s first episode of TdP and VF was considered to be associated with the administration of moxifloxacin. Amiodarone, a class III antiarrhythmic, prolongs myocardial repolarization and the QT interval by suppressing automaticity in the sinoatrial and atrioventricular nodes (14). Although the TdP risk with amiodarone is relatively low, co-administration with other QT-prolonging agents necessitates vigilant rhythm monitoring (8, 15). The second episode of refractory TdP and VF was considered to be related to the subsequent use of amiodarone in the setting of an already prolonged QTc interval. Therefore, although amiodarone is a standard drug recommended by ACLS guidelines, the QTc interval must be checked before its use, especially when ventricular tachycardia is the presenting rhythm of the cardiac arrest. According to expert consensus on catheter ablation for ventricular arrhythmias (16), the first-line treatment for recurrent TdP is intravenous magnesium sulfate. In cases of bradycardia-associated TdP, temporary pacing or agents such as isoproterenol can be used to increase heart rate. Electrical defibrillation is indicated in hemodynamically unstable TdP or progression to VF. For patients unresponsive or intolerant to standard therapy, alternative treatment strategies must be considered.

Lidocaine, a class Ib antiarrhythmic agent, exerts its effects by blocking sodium channels, shortening action potential duration, and increasing the refractory period. It is widely used for ventricular premature beats, ventricular tachycardia, and VF (17). Continuous intravenous infusion of lidocaine has been shown to significantly reduce QT-prolongation-related arrhythmic events (18). Anisodamine, an anticholinergic alkaloid, modulates vascular endothelial function, relieves coronary and microvascular spasms, and has been shown to improve microcirculatory perfusion in septic shock (19). The hyperinflammatory state and elevated catecholamine levels in septic shock increase the risk of coronary vasospasm, which anisodamine can effectively alleviate (6). Moreover, studies have found that anisodamine alleviated myocardial damage in septic shock rats by inhibiting the NF-κB/NLRP-3 pathway and promoting the PI3K-AKT pathway, thereby mitigating inflammation and cell apoptosis (20). However, the potential synergistic effect of anisodamine with lidocaine remains unclear, and its exact contribution warrants further investigation. In the present case, an 89-year-old male with septic shock (positive blood culture, SOFA score of 17) developed SCM characterized by markedly reduced papillary muscle movement and an LVEF of 40%, accompanied by elevated troponin, myoglobin, and BNP levels. After receiving moxifloxacin and amiodarone, his QTc prolonged to 647 ms, which was likely attributable to the combined effects of sepsis, advanced age, and these medications, eventually leading to refractory TdP and VF. Standard interventions including defibrillation, magnesium and potassium supplementation were ineffective. However, intravenous lidocaine combined with anisodamine successfully suppressed the malignant arrhythmias, and the frequency of ventricular ectopy and nonsustained ventricular tachycardia was significantly reduced. By day 14, echocardiogram confirmed restoration of normal wall motion and improved cardiac function. Coronary CTA revealed no obstructive coronary disease. Overall, this case was consistent with septic cardiomyopathy, although the diagnosis remains one of exclusion and lacks standardized criteria—an area warranting further research.

## Conclusion

Moxifloxacin and amiodarone can exacerbate QTc prolongation in patients with septic cardiomyopathy, leading to refractory torsades de pointes and ventricular fibrillation. In addition to standard treatments such as defibrillation, electrolyte correction, and resuscitation, intravenous lidocaine combined with anisodamine may represent a potential therapeutic option for reversing malignant arrhythmias, though this finding requires validation in larger studies. In elderly patients with septic shock, early cardiac ultrasound and dynamic QT/QTc monitoring are recommended, and the use of QT-prolonging drugs should be avoided whenever possible to prevent life-threatening arrhythmias.

## Data Availability

The original contributions presented in this study are included in this article/supplementary material, further inquiries can be directed to the corresponding author.
